# A real-world stepped wedge cluster randomized trial of practice facilitation to improve cardiovascular care

**DOI:** 10.1186/s13012-015-0341-y

**Published:** 2015-10-28

**Authors:** Clare Liddy, William Hogg, Jatinderpreet Singh, Monica Taljaard, Grant Russell, Catherine Deri Armstrong, Ayub Akbari, Simone Dahrouge, Jeremy M. Grimshaw

**Affiliations:** C.T. Lamont Primary Health Care Research Centre, Bruyère Research Institute, Ottawa, Ontario Canada; Department of Family Medicine, University of Ottawa, Ottawa, Ontario Canada; Clinical Epidemiology Program, Ottawa Hospital Research Institute, Ottawa, Ontario Canada; Department of Epidemiology and Community Medicine, University of Ottawa, Ontario, Canada; Southern Academic Primary Care Research Unit, School of Primary Health Care, Monash University, Notting Hill, Victoria Australia; Department of Economics, University of Ottawa, Ottawa, Ontario Canada; The Ottawa Hospital, Ottawa, Ontario Canada; Department of Medicine, University of Ottawa, Ottawa, Ontario Canada; Bruyère Research Institute, 43 Bruyère St, Annex E, Room 106, Ottawa, Ontario K1N 5C8 Canada

**Keywords:** Practice facilitation, Primary care, Cardiovascular health

## Abstract

**Background:**

Practice facilitation has been associated with meaningful improvements in disease prevention and quality of patient care. Using practice facilitation, the Improved Delivery of Cardiovascular Care (IDOCC) project aimed to improve the delivery of evidence-based cardiovascular care in primary care practices across a large health region. Our goal was to evaluate IDOCC’s impact on adherence to processes of care delivery.

**Methods:**

A pragmatic stepped wedge cluster randomized trial recruiting primary care providers in practices located in Eastern Ontario, Canada (ClinicalTrials.gov: NCT00574808). Participants were randomly assigned by region to one of three steps. Practice facilitators were intended to visit practices every 3–4 (year 1—intensive) or 6–12 weeks (year 2—sustainability) to support changes in practice behavior. The primary outcome was mean adherence to indicators of evidence-based care measured at the patient level. Adherence was assessed by chart review of a randomly selected cohort of 66 patients per practice in each pre-intervention year, as well as in year 1 and year 2 post-intervention.

**Results:**

Eighty-four practices (182 physicians) participated. On average, facilitators had 6.6 (min: 2, max: 11) face-to-face visits with practices in year 1 and 2.5 (min: 0 max: 10) visits in year 2. We collected chart data from 5292 patients. After adjustment for patient and provider characteristics, there was a 1.9 % (95 % confidence interval (CI): −2.9 to −0.9 %) and 4.2 % (95 % CI: −5.7 to −2.6 %) absolute decrease in mean adherence from baseline to intensive and sustainability years, respectively.

**Conclusions:**

IDOCC did not improve adherence to best-practice guidelines. Our results showed a small statistically significant decrease in mean adherence of questionable clinical significance. Potential reasons for this result include implementation challenges, competing priorities in practices, a broad focus on multiple chronic disease indicators, and use of an overall index of adherence. These results contrast with findings from previously reported facilitation trials and highlight the complexities and challenges of translating research findings into clinical practice.

**Trial registration:**

ClinicalTrials.gov NCT00574808

**Electronic supplementary material:**

The online version of this article (doi:10.1186/s13012-015-0341-y) contains supplementary material, which is available to authorized users.

## Introduction

Practice facilitation is an approach to implementing evidence-based best practices in primary care [1]. It involves bringing an external healthcare professional (often a trained nurse with management experience) into a practice, in order to help primary care providers address the challenges associated with implementing evidence-based guidelines. Practice facilitators (also known as outreach facilitators, practice enhancement assistants, and practice coaches) are trained experts in initiating change in practices. They work with practices to identify areas for improvement, set care improvement goals, and provide tools and approaches to reach these goals. Facilitation has been found to lead to improvements in prevention, diabetes care, smoking cessation, and cancer care [2–4]. A recent meta-analysis demonstrated that primary care providers are more likely to adopt evidence-based guidelines when supported by a facilitator [5]. Furthermore, a cost consequence analysis of a facilitation study that focused on improving preventive care in primary care practices demonstrated a 40 % return on intervention investment [6].

Facilitation programs are being broadly implemented by practice-based research networks, health departments, professional associations, and health plans. As such, ongoing evaluation is needed to determine the effectiveness of practice facilitation in real-world settings.

The adoption, optimal intensity, and duration of practice facilitation remain uncertain, as does the effectiveness beyond single-disease-focused facilitation programs [1, 2, 5, 7–9].

We initiated the Improved Delivery of Cardiovascular Care (IDOCC) through an outreach facilitation project in 2007 to improve adherence to guidelines for the secondary prevention of heart disease, stroke, peripheral vascular disease, renal disease, and diabetes in primary care practices in a health region [10]. We hypothesized that facilitation would enable practices to improve overall adherence to cardiovascular care guidelines. This paper reports on our primary outcome of adherence to guidelines as measured by mean adherence to care delivery in the practice, based on patient-level data.

## Methods

The IDOCC study protocol has been published elsewhere [10], so here, we provide a brief overview of the methods as per CONSORT [11].

### Setting and participants

We conducted the study in a large health region in Eastern Ontario, Canada (16,000 km^2^), including Ottawa and the surrounding rural communities. It is a culturally diverse region of 1.2 million individuals who have chronic disease burdens and patient health outcomes comparable to Ontario and the rest of Canada [12].

Practices were eligible to participate if they provided general primary care services and had been in operation for at least 2 years prior to the initiation of the intervention. We enlisted practices if at least one physician from that practice consented to participate. Physicians received no monetary compensation for participating but were eligible for continuing professional development credits with the College of Family Physicians of Canada.

### Design

**Fig. 1:**
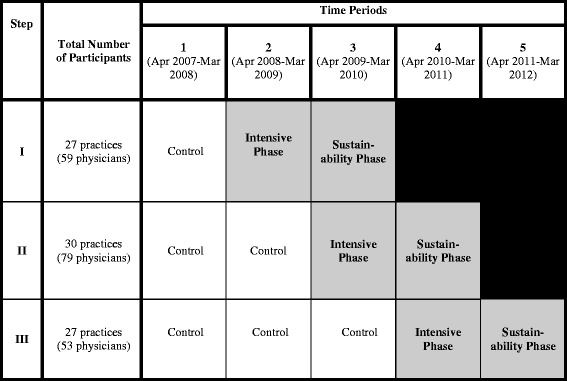
IDOCC stepped wedge study design. Shaded cells represent intervention periods.  Blank cells represent control periods

We used a stepped wedge cluster randomized trial design (see Fig. 1) in which the intervention was sequentially rolled out to participating primary care practices assigned by region in three steps. Each step provides data for both control and intervention periods, and data analysis proceeds by comparing time points across steps in control versus intervention periods.

We chose the stepped wedge design to (1) minimize the practical, logistical, and financial constraints associated with large-scale project implementation, (2) control for the effect of time, and (3) ensure that all practices in the project were eventually offered the intervention [13]. The Ottawa Hospital Research Ethics Board approved this trial (2007292-01H).

### Randomization

We divided the health region systematically into nine geographic divisions using geographic information system mapping technology [14], stratifying the divisions by their location within the region (i.e., west, central, and east). We randomly assigned each of the three divisions per stratum (i.e., west, central, or east) to one of three study steps using computer-generated random numbers provided by an independent statistician. Each step comprised three divisions, with each step having a division from the east, central, and west part of the region. As such, each division per stratum had the same probability of beginning the program at any given step.

We developed a list, updated prior to recruitment at each step, of the contact information of all physicians practicing in the geographic regions of interest using a variety of physician listings such as The College of Physicians and Surgeons of Ontario website [15], the public telephone directory, a provincial directory of group practices, and through direct contact with the practices. We used a modified Dillman approach to recruit practices. This involved sending reminders and repeat mailings from the study team [16]. Recruitment continued in each step until we reached the desired sample. We obtained consent from all participating physicians.

### Intervention

The intervention consisted of regular meetings with a facilitator over the study period. Practice facilitators intended to visit with practices 13 times (i.e., one visit every 4 weeks) in year 1 (intensive phase) and then 4–6 times (i.e., one visit every 8–12 weeks) in year 2 (sustainability phase). Starting with audit and feedback, consensus building and goal setting, the facilitators supported practices in changing their behavior through the incorporation of the following chronic care model elements: (1) integrated evidence-based care guideline and other decision support tools, (2) enhanced community linkages, (3) self-management support, and (4) delivery system redesign such as introducing a practice population approach, recall systems, and disease-specific registries. Facilitators encouraged practices to implement small but continuous changes through the plan-do-study-act cycle, which is a common quality improvement tool [17]. Full details of the intervention have been published previously [10] and are available in Additional file 1.

### Outcome

The primary outcome for the trial was a patient-level score intended to reflect adherence to recommended guidelines for cardiovascular disease processes of care. The score represented the percentage of recommended process of care indicators for which the patient was eligible that were performed on the patient over a 1-year period. The indicators were (1) two blood pressure measures, (2) lipid profile, (3) waistline measure, (4) smoking status, (5) glycemic levels (two hemoglobin A1c measures for patients with diabetes or one fasting blood glucose for high-risk patients without diabetes), (6) kidney function (albumin-to-creatinine ratio or estimated glomerular filtration rate), (7) prescription of all eligible medications, and (8) referral to a smoking cessation program. For each step, we measured mean adherence for baseline time points and for the intensive (year 1) and sustainability (year 2) phases.

### Data collection

The unit of intervention was the practice, but the unit of analysis and causal inference was the patient. Causal inference refers to the fact that although the intervention is aimed at producing a system-level change in the practice, the assessment of this change will occur at the patient level through the review of medical charts from the practice. For each practice, we randomly selected 66 charts of patients who were over age 40 and (1) had cardiovascular disease including coronary artery disease, cerebrovascular disease (stroke and/or transient ischemic attack), or peripheral vascular disease; (2) had diabetes or chronic kidney disease; or (3) were at high risk of developing cardiovascular disease as defined by age (males ≥45, females ≥55), smoking status, hypertension, weight, or dyslipidemia. The same patients were followed over time to assess outcomes.

We collected data for each time block in the stepped wedge design as highlighted in Fig. 1. We used the baseline data collection over 1 year from each practice for the audit and feedback stage of the facilitator intervention. In total, step I practices provided 3 years of data (1 year of baseline data, intensive year, and sustainability year), step II practices provided 4 years of data (2 years of baseline data, intensive year, and sustainability year), and step III practices provided 5 years of data (3 years of baseline data, intensive year, and sustainability year).

Six trained nurses completed the chart abstraction. A four-part quality implementation and monitoring process was used to ensure consistent levels of data quality across chart abstractors. Full details of our chart abstraction methods are described elsewhere [18].

We collected practice-level data through practice surveys and by linking family physicians’ medical ID to their demographic and practice model information contained in the Institute for Clinical Evaluative Sciences’ Corporate Provider Database, which reflects characteristics as of March 31, 2008 [17, 19].

The chart abstractors, facilitators, and practices were blinded to the full details of which data were being collected for the primary outcome analysis, and chart abstractors were additionally blinded as to whether the practice they were auditing was in the control or intervention phase.

### Sample size calculation

There are as yet no published sample size formulae for a stepped wedge trial with a cohort design. Using the sample size calculation formula for a two-arm parallel design, we determined that 21 practices in each of the intervention and control arms with an average of 66 patients per practice (the anticipated average number of eligible patients per practice based on pilot data) would detect an 8 % absolute mean difference (deemed clinically relevant) in the mean adherence score using a two-sided test at the 5 % level of significance with 90 % power. This difference was deemed clinically relevant after conducting a series of simulations followed by discussions with a panel of family physicians and cardiologists. Our calculations assumed an intra-cluster correlation coefficient of 0.18 and a common standard deviation of 18 % calculated using IDOCC pilot data on nearly 500 patients across seven practices. Our target sample size was 30 practices in each step of the stepped wedge design to account for 20 % potential attrition. Since the stepped wedge design includes repeated observation periods per practice and is therefore more powerful than the simple parallel design, this sample size estimate was considered to be conservative [20].

### Data analysis

We generated descriptive statistics summarizing practice and patient characteristics for the entire sample and for each step separately. We used general linear mixed-effect regression modeling as described by Hussey and Hughes to assess the impact of the IDOCC program on the primary outcome [21]. To assess the normality of the distribution of the primary outcome, we used visual inspection of histograms.

The model included fixed effects for time, treatment phase, and region and a random effect for the practice. Regions were treated as fixed—rather than as random—effects as they could not be considered a sample from some population but reflected a systematic division of Eastern Ontario. In addition, a compound symmetric covariance structure was specified to account for the correlation in repeated measures on the same patient over time. We estimated the model using restricted maximum likelihood. In the analysis, the treatment variable was specified as a three-level categorical variable (baseline, intensive, sustainability).

We conducted an unadjusted analysis (model A) followed by two adjusted analyses: model B adjusted for patient characteristics (age, sex, median neighborhood income, number of cardiovascular-related diseases, number of cardiovascular-related risk factors, and rurality) [22, 23], while model C included three practice-level characteristics that have been shown to influence primary care practice adherence to guidelines (payment model, percentage of participating physicians that were female, and average number of years since graduation (to 2010)) [24, 25]. We carried out pairwise comparisons between mean adherence scores across the different treatment years (baseline, intensive year, sustainability year) at the 0.017 Bonferroni-corrected level to maintain the familywise error rate at 5 %. We calculated adjusted least square mean adherence scores in each phase by setting continuous covariate values equal to their mean values and using observed marginal distributions for categorical variables. Thus, least square mean differences represent the estimated effect of the intervention on the quality of care for an “average” patient in an “average” practice. All analyses were conducted using SAS, Version 9.3, SAS Institute Inc.

## Results

We approached all 533 primary care practices in the Champlain region to participate in the IDOCC program. Ninety-nine were ineligible to participate because the practice was no longer in operation, the clinic was an exclusive walk-in practice, or the physician running the practice was planning to retire within the 2-year intervention time frame. Of the 434 eligible practices, 93 practices (21 %) (194 physicians) agreed to participate. Nine practices withdrew from the study prior to the initiation of the facilitation intervention for various reasons, including not having the office space to accommodate a chart abstractor and insufficient number of eligible high-risk patient charts, leaving 84 participating practices (182 physicians): 27, 30, and 27 in steps I, II, and III, respectively. The withdrawal of ten practices from the study resulted in a loss of only 12 physicians, suggesting that only small or single physician practices chose to withdraw. The first facilitation visit was on April 14, 2008, and the final site visit was March 27, 2012. The trial concluded as scheduled at the end of the intervention phase for step III. No practices withdrew after the start of the intervention.

Baseline characteristics of participating practices, providers, and patients are presented in Table 1. The majority of participating practices were fee-for-service (53.5 %) and located in urban areas (82.1 %).

**Table 1:** Breakdown of practice-, provider-, and patient-level characteristics at baseline by step

Characteristic	Step I	Step II	Step III	Overall
Practice level				
Number of practices (*n*)	27	30	27	84
Primary care model (*n*, %)				
Fee for service	22 (81.5 %)	12 (40.0 %)	11 (40.7 %)	45 (53.5 %)
Capitation—non-interdisciplinary	1 (3.7 %)	8 (26.7 %)	11 (40.7 %)	20 (23.8 %)
Capitation—interdisciplinary	0 (0.0 %)	4 (13.3 %)	3 (11.1 %)	7 (8.3 %)
Community health centers	4 (14.8 %)	6 (20.0 %)	2 (7.4 %)	12 (14.3 %)
Rural practices (*n*, %)	4 (14.8 %)	4 (13.3 %)	7 (25.9 %)	15 (17.9 %)
Baseline adherence score (mean, SD)	0.62 (0.20)	0.65 (0.22)	0.64 (0.22)	0.64 (0.22)
Provider level				
Number of providers (*n*)	57	78	47	182
Female (*n*, %)	34 (59.7 %)	51 (65.4 %)	27 (57.5 %)	112 (61.5 %)
Years since graduation from 2010 (SD)	27 (10)	23 (7)	25 (10)	25 (9)
Patient level				
Number of patients (*n*)	1667	1891	1734	5292
Age (mean, SD)	68 (13)	66 (12)	67 (12)	66 (12)
Female (*n*, %)	852 (51.1 %)	1003 (53.0 %)	851 (49.1 %)	2706 (51.1 %)
Number of cardiovascular-related conditions^a^ (mean, SD)	1.18 (0.91)	1.17 (0.95)	1.26 (0.97)	1.20 (0.95)
Number of cardiovascular risk factors^b^ (mean, SD)	2.67 (0.81)	2.82 (0.68)	2.79 (0.74)	2.76 (0.75)
Rural residents (*n*, %)	436 (26.2 %)	440 (23.3 %)	774 (44.6 %)	1650 (31.2 %)

^a^Cardiovascular-related diseases examined: stroke, coronary artery disease, peripheral vascular disease, diabetes, chronic kidney disease

^b^Risk factors assessed were age, hypertension, dyslipidemia, and smoking

We collected baseline data from 5292 patient medical charts, of which 627 (11.8 %) were lost at follow-up, primarily due to patients dying or moving within the study period. Chart abstraction data quality assessments via re-abstractions demonstrated an overall inter-rater reliability kappa value of 0.91 and a percent agreement of 93.9 %.

No practices received the intended intensity of facilitator visits. Only 32 practices had eight or more visits over 2 years. On average, facilitators had 6.6 (min: 2 max: 11) face-to-face visits with practices in year 1 and 2.5 (min: 0 max: 10) visits in year 2. In addition to face-to-face visits, facilitators communicated with practices an average of 1.7 times using phone or email in year 1 and 1.6 times in year 2.

**Fig. 2:**
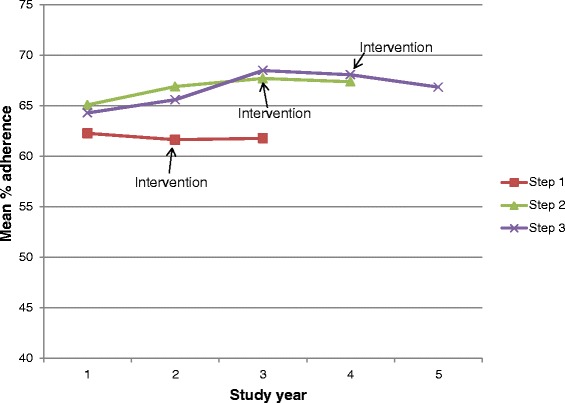
Observed mean adherence score (%) over time in practices allocated to study steps, indicating year of implementation of the intervention

**Fig. 3:**
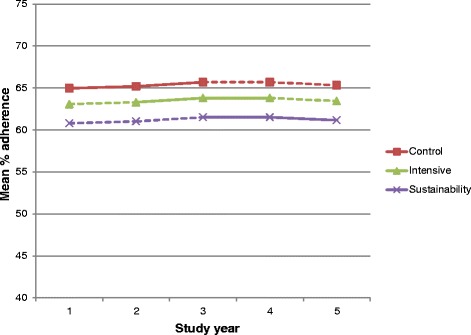
Estimated effect of the IDOCC intervention presented as adjusted mean adherence score (%) averaged over all practices in control, intensive, and sustainability conditions. The model assumes a single underlying secular trend across steps and estimates a shift in level as a result of the intervention. *Solid lines* represent time intervals with observed data in that condition; *dashed lines* represent time intervals with no observed data in that condition

Figure 2 presents the observed mean adherence scores for each study step during the baseline, intensive, and sustainability years. There is little change in the mean adherence score pre- and post-intervention for all practices in all three steps. Additionally, the three baseline time points for step III appear to show a (modest) upward secular trend in mean adherence, highlighting the importance of controlling for the time trend in the subsequent regression analysis. Fig. 3 presents the estimated effect of the IDOCC intervention presented as adjusted mean adherence score averaged over all practices in control, intensive, and sustainability conditions.

Using data from all three steps, the unadjusted (model A) least square mean differences showed an absolute decrease from baseline of 2.1 % in the intensive phase (95 % CI: −3.1 to −1.1 %) and a decrease of 4.7 % in the sustainability phase (95 % CI: −6.2 to −3.2 %) (see Table 2). After adjustment for patient characteristics (model B), there was an absolute decrease in mean adherence from baseline of 1.9 % in the intensive phase (95 % CI: −2.9 to −0.9 %) and a decrease of 4.2 % in the sustainability phase (95 % CI: −5.7 to −2.6 %). Adjusting for provider factors had little impact on these estimates.

**Table 2:** Results from multivariable mixed-effect regression analyses of the effect of the IDOCC intervention

Parameter	Model A			Model B			Model C		
	(Unadjusted)			(Adjusted for patient factors)			(Adjusted for patient/provider factors)		
	Estimate^a^	*P* value	95 % CI	Estimate^a^	*P* value	95 % CI	Estimate^a^	*P* value	95 % CI
Intercept	61.4	<0.0001	56.7 to 66.1	39.2	<0.0001	32.6 to 45.9	44.97	<0.0001	34.8 to 55.1
Time									
Year 1	Ref	-	-	Ref	-	-	Ref	-	-
Year 2	1.5	<0.0001	0.8 to 2.3	0.9	0.02	0.2 to 1.7	0.9	0.019	0.2 to 1.7
Year 3	4.2	<0.0001	3.2 to 5.3	3.0	<0.0001	1.9 to 4.0	3.0	<0.0001	1.9 to 4.0
Year 4	6.3	<0.0001	4.6 to 7.9	4.4	<0.0001	2.8 to 6.0	4.4	<0.0001	2.8 to 6.0
Year 5	7.3	<0.0001	5.1 to 9.5	4.9	<0.0001	0.027 to 0.071	4.9	<0.0001	2.7 to 7.1
Intervention phase									
Baseline	Ref	-	-	Ref	-	-	Ref	-	-
Intensive	−2.1	<0.0001	−3.2 to −1.1	−1.9	0.0003	−2.9 to −0.9	−1.9	0.0003	−2.9 to −0.9
Sustainability	−4.7	<0.0001	−6.2 to −3.2	−4.2	<0.0001	−5.7 to −2.6	−4.2	<0.0001	−5.7 to −2.6
Region									
Region 1	Ref	-	-	Ref	-	-	1.0	0.789	−6.6 to 8.7
Region 2	1.1	0.756	−5.9 to 8.1	1.4	0.695	−5.5 to 8.3	4.1	0.25	−2.9 to 11.0
Region 3	3.8	0.205	−2.1 to 9.7	3.5	0.24	−2.3 to 9.3	2.7	0.531	−5.7 to 11.0
Region 4	2.4	0.535	−5.3 to 10.2	2.3	0.545	−5.3 to 9.9	0.2	0.956	−6.7 to 7.1
Region 5	2.4	0.441	−3.6 to 8.3	0.8	0.780	−5.1 to 6.8	3.7	0.293	−3.2 to 10.6
Region 6	4.5	0.157	−1.7 to 10.7	3.7	0.231	−2.4 to 9.9	5.4	0.184	−2.6 13.4
Region 7	5.7	0.121	−1.5 to 13.0	5.7	0.116	−1.4 to 12.9	Ref	-	-
Region 8	−1.3	0.745	−9.0 to 6.4	−1.0	0.798	−8.6 to 6.6	4.8	0.189	−2.4 to 12.1
Region 9	2.3	0.454	−3.7 to 8.3	2.2	0.476	−3.9 to 8.1	2.1	0.576	−5.1 to 9.2
Patient level									
Age				0.03	0.168	−0.01 to 0.06	0.03	0.14	−0.009 to 0.07
Sex				0.09	0.825	−0.7 to 0.9	0.07	0.872	−0.8 to 0.9
Income				0.39	0.004	0.1 to 0.7	0.4	0.0035	0.13 to 0.67
# of cardiovascxular-related diseases^b^									
0 (risk factors Only)				Ref	-	-	Ref	-	-
1				4.8	<0.0001	3.9 to 5.8	4.8	<0.0001	3.9 to 5.8
2				6.9	<0.0001	5.7 to 8.0	6.9	<0.0001	5.7 to 8.0
3				6.6	<0.0001	5.1 to 8.1	6.6	<0.0001	5.1 to 8.2
4				7.4	<0.0001	4.9 to 9.9	7.4	<0.0001	4.9 to 9.9
5				11.0	0.001	4.3 to 17.6	11.0	0.0012	4.4 to 17.6
# of risk factors for cardiovascular disease^c^									
0				Ref	-	-	Ref	-	-
1				6.5	0.0009	2.6 to 10.3	6.5	0.0009	2.6 to 10.3
2				12.7	<0.0001	9.0 to 16.4	12.7	<0.0001	8.9 to 16.4
3				17.1	<0.0001	13.4 to 20.9	17.1	<0.0001	13.4 to 20.9
4				4.7	0.017	0.8 to 8.5	4.6	0.019	0.8 to 8.5
Rural				0.0099	0.103	−0.2 to 2.2	0. 9	0.141	−0. 3 to 2.1
Provider level									
Payment model									
FFS							Ref	-	-
Capitation							1.4	0.437	−2.1 to 4.9
Salary							9.0	0.710	−3.7 to 5.5
Female physician							1.7	0.320	−1.6 to 5.0
Years since graduation (as of 2010)							−0.27	0.0015	−0.4 to −0.1

^a^Negative estimate indicates a decrease in adherence

^b^Cardiovascular-related diseases examined: stroke, coronary artery disease, peripheral vascular disease, diabetes, chronic kidney disease

^c^Risk factors assessed were age, hypertension, dyslipidemia, and smoking

## Discussion

In our pragmatic trial of practice facilitation, the intervention did not improve adherence to evidence-based guidelines for cardiovascular disease in primary care practices. These results contrast with findings from previously reported facilitation trials [5, 26–28] and highlight the complexities and challenges of scaling up research studies into sustainable programs. Suboptimal intensity of the intervention, a broad focus on multiple chronic conditions, and measurement challenges are all factors that may have contributed to the null results.

A meta-analysis conducted of facilitation programs demonstrated that intensity as measured by number of facilitation visits was associated with greater effect size [5]. The IDOCC intervention was designed with the intent of having the facilitators meet face-to-face with practices 13 times (i.e., one visit every 4 weeks) in year 1 (intensive phase) and 4–6 times in year 2 during the sustainability phase [29, 30]. We were unable to attain this “dose” with our practices, who despite having the best intentions of engaging and meeting with the facilitators, were all unable to find the time for this frequency of meetings. Other facilitation studies have faced similar issues. For instance, facilitators in a group-randomized practice facilitation trial conducted in South Texas initially intended to make monthly facilitation visits over 12 months but due to competing demands within participating practices (e.g., EMR implementation, staff turnover) were only able to make six to seven visits during the 1-year study period [31]. The difficulties in achieving the requisite intensity of visits necessary to facilitate changes suggest that practice facilitation may not be as effective in complex, real-world settings. In our program, no incentives were provided apart from the opportunity to claim continuing professional development credits. Supporting practice change may require incentives to compensate providers for their time and/or policies designed to encourage a culture of quality improvement such as mandatory reporting of quality metrics and accreditation of primary care practices. Additionally, financial incentives have been identified as having a modest positive impact on primary care processes associated with quality management of chronic disease [32].

Improving quality of care delivery for people with multimorbidity is an area that requires more research. Although our study was grounded in the chronic care model approach with targeted practice level improvement activities across several dimensions within the model, it failed to improve care delivery. Previous facilitation studies primarily focused on single diseases such as diabetes and asthma [5, 33, 34], whereas our intervention attempted to improve adherence to guidelines for patients with a broad number of cardiovascular-related conditions (coronary artery disease, peripheral vascular disease, stroke/TIA, diabetes, and chronic kidney disease) and risk factors (hypertension, dyslipidemia, weight, and smoking). Implementing guidelines for people with multiple chronic conditions is complex. While physicians may be able to successfully focus on single diseases such as diabetes or asthma, improving adherence across multiple conditions is more challenging. A review of interventions for managing patients with multimorbidity found that programs targeting specific areas of concern for the patient (e.g., functional difficulties) were more effective than broader disease-oriented programs [35]. Likewise, recent studies (including ours) examining the implementation of practice facilitation programs targeting multiple diseases have found only modest or insignificant improvements in patient outcomes [31, 33].

Our analyses identified statistically significant decreases in our mean adherence score; however, these changes are likely the result of our study being overpowered rather than the impact of the program on clinical behavior. The decreases we detected were smaller than our pre-specified minimal clinical difference and hence unlikely to have any clinical significance. We chose to measure adherence to all indicators using a mean adherence score as we felt it would reflect the reality of managing multiple chronic conditions simultaneously. We hypothesized that practice facilitation’s focus on practice-level transformation would lead to changes, which would positively influence multiple processes of care and could be captured with a mean score. However, we were unable to demonstrate this. Measuring impact across multiple areas is challenging. Our adherence score was an unweighted mean in which all indicators were considered equally important. As a result, it is more difficult to interpret the clinical importance of observed changes: whereas a decrease of 2–4 % could represent deterioration in one to two individual indicators if practices improved in some areas but deteriorated in others, such changes would not be detectable. For these reasons, future analyses are being planned of the individual indicators of adherence.

### Strengths and limitations

Our study had several strengths, including our population approach and implementation across a large geographic area. We had a large sample size that included a diverse range of practices. Our stepped wedge design addressed the need of our funders to eventually offer this practice improvement program to all participants. Our low rate of attrition speaks to the feasibility of establishing ongoing relationships with the practices despite competing demands. Although practices that agreed to participate may not be representative of all practices across Eastern Ontario, they represent the kinds of practices expected to participate in QI initiatives in a real-world setting. Moreover, in this randomized controlled trial, internal validity is of primary concern.

Limitations include the use of an index of adherence, which provides an overall picture of change in adherence patterns but may hide certain changes that may have occurred during the intervention. We had also considered assigning specific weights to each indicator in order to reflect their relative importance to patient health outcomes. Unfortunately, there is no empirical evidence for assigning weighting factors to the indicators in this study. Although theoretically useful, many authors have acknowledged the difficulty in effectively determining meaningful weights, and as such, few researchers use this approach [36]. Also, due to practical and logistical constraints associated with implementing the intervention across a large geographic region, we were unable to randomize individual practices. As a result of randomizing at a regional level, there were some imbalances in the characteristics of participants across the three steps, as well as differences in the secular trend across steps. To address these limitations, we adjusted for observed differences in the analysis and examined models that included interactions with each step.

## Conclusion

Our findings highlight the complexities of expanding research evidence into effective, sustainable programs. Whether practices can commit the time required without policy or incentives is unknown. Further research is needed to better understand what impact practice facilitation can have on eliciting change at this level once implemented more broadly.

## Additional files

Additional file 1:
**The Improved Delivery of Cardiovascular Care (IDOCC) Intervention.** The file contains the full details of the intervention. Additional file 2:
**CONSORT 2010 checklist.** This is a checklist of the information to include when reporting a cluster randomized trial.

## Abbreviations

IDOCC: Improved Delivery of Cardiovascular Care
