# Supplementary material for: A real-world stepped wedge cluster randomized trial of practice facilitation to improve cardiovascular care
Source: Implement Sci. 2015 Oct 28;10:150. doi: 10.1186/s13012-015-0341-y (PMC4625868; doi:10.1186/s13012-015-0341-y)
Supplement: Additional file 1: — The Improved Delivery of Cardiovascular Care (IDOCC) Intervention. The file contains the full details of the intervention. [file 13012_2015_341_MOESM1_ESM.docx]

**Appendix A:** The Improved Delivery of Cardiovascular Care (IDOCC) Intervention

The intervention began with an audit to assess each practice’s pre-intervention performance in four areas: 1) disease/risk factor screening, 2) drug prescribing patterns, 3) use of external resources (e.g., diabetes clinics, smoking cessation programs), and 4) patient group health status (i.e., patients at target for clinical tests such as blood pressure). After providing performance feedback to the practice, the practice facilitator (PF) worked with the practice to identify goals for improving their delivery of evidence-based cardiovascular care. PFs met regularly with practices and used a multifaceted approach to assist them in incorporating elements of the chronic care model into daily practice routines, better adhering to evidence-based guidelines, and reaching their specific goals. These elements included: a) evidence-based decision support for providers using an integrated CVD guideline, b) delivery system redesign for practices, c) enhanced self-management support tools provided to practices to help them engage patients, and d) increased community resource linkages for practices to enhance referral of patients.

*a) Decision Support:* Decision support was guided primarily by The Champlain Cardiovascular Disease Prevention and Management Guideline (www.idocc.ca). The goal of the guideline was to harmonize the management and target outcomes for multiple vascular conditions (Coronary Artery Disease, TIA/Stroke, Diabetes, Renal failure and Peripheral Vascular Disease), summarize evidence-based strategies for the detection and management of these vascular conditions and their associated risk factors (high blood pressure, high cholesterol, smoking, physical inactivity, and obesity), and maximize the use of local resources and tools in the provision of care. This guideline is a very valuable tool, as most primary care physicians struggled to follow multiple, sometimes conflicting guidelines for each individual cardiovascular condition or risk factor. [1,2] The Champlain CVD Prevention and Management Guideline is the first Canadian guideline for primary care which includes standardized care pathways for patients with multiple chronic disease and cardiovascular risk factors. It was developed based on the recommendations of seven Evidence Monitoring Committees established for each of the seven risk factors targeted by the IDOCC project: hypertension, dyslipidemia, diabetes, chronic kidney disease, smoking, obesity, and physical inactivity.[3]

*b) Delivery system re-design:* In helping physicians reach their goals, PFs focus on assisting practices to set up new systems and processes to help improve care delivery. Specific examples of improvement strategies include the utilization of registries to track patients with certain conditions, recall systems, reminder systems, and group visits for patients with common conditions (e.g., diabetes patients come in at one time to have screening tests performed and to learn about self-management)

*c) Self-management support tools:* The IDOCC project developed an inventory of self-management support tools such as pocket cards, flow sheets, questionnaires, and patient self-management action plan forms to help physicians improve their delivery of evidence-based care. Tools can be accessed at http://www.idocc.ca/en_toolkits.php.

*d) Community resources:* A key feature of the Champlain Cardiovascular Disease Prevention and Management Guideline is the community resource section, which lists all programs relevant to cardiovascular care for a given condition or risk factor. For example, current community smoking cessation programs and exercise programs are listed with referral information specific to each region - this information was kept up to date by both the work of the PFs and linkages established within the Champlain Cardiovascular Disease Prevention Network. An annual update was done on the online version of the guideline.[4] The PFs also connect with the education and community programs directly and provide key information to the practices based on stated needs.

*e) Program tailoring:* The IDOCC facilitation approach was tailored to meet the specific goals and needs of each practice. For example, a practice that discovers through their audit and feedback that the rate of smokers in their high risk group exceeds 25% as compared to an average of 13% for the region, may then choose to focus their improvement efforts on smoking cessation. The PF will then introduce providers to the available tools, resources, and possible practice redesign techniques that could be incorporated into regular practice. Another practice may choose to focus on increasing referral to weight management programs or improving management of their patients with hypertension. In each case, the practice determines its specific improvement goals and targets and the facilitators assist them in achieving their goals.

References:

1. Epping-Jordan JE, Pruitt SD, Bengoa R, Wagner E. Improving the quality of health care for chronic conditions. Qual Saf Health Care. 2004;13.(4):299-305.
2. Hensrud DD. Clinical preventive medicine in primary care: background and practice: 1. Rationale and current preventive practices. Mayo Clin Proc. 2000;75(2):165-72.
3. Montoya L, Liddy C, Hogg W, Papadakis S, Dojeiji L, Russell G, et al. Development of the Champlain primary care cardiovascular disease prevention and management guideline: tailoring evidence to community practice. Can Fam Physician. 2011;57(6):e202-7.
4. Champlain Cardiovascular Disease Prevention Network. The Champlain Primary Care Cardiovascular Disease Prevention and Management Guideline. Champlain LHIN. 2012. http://ccpnetwork.ca/wp-content/uploads/2013/05/Guideline2012.pdf. Accessed 7 May 2015.
